# The multiparametric prostate resonance imaging with the prostate imaging-reporting and data system (PI-RADS): the state of art of prostate cancer diagnosis

**DOI:** 10.1590/S1677-5538.IBJU.2019.03.01

**Published:** 2019-07-27

**Affiliations:** 1 Unidade de Pesquisa Urogenital da Univ. Estadual do Rio de Janeiro – UERJ, Rio de Janeiro, RJ, Brasil; 2 Hospital Federal da Lagoa, Rio de Janeiro, RJ, Brasil; 3 Editor Associado da International Braz J Urol

The May-June 2019 issue of the International Brazilian Journal of Urology presents original contributions with a lot of interesting papers in different fields: Prostate Cancer, Renal stones, Renal Cell Carcinoma, Bladder Cancer, Prostate Biopsy, Kidney Transplant, Neurogenic Bladder and Upper Urinary tract urothelial carcinoma. The papers come from many different countries such as Brazil, USA, Turkey, China, India, Taiwan, Spain, Poland, Japan, Portugal, Israel and United Kingdon, and as usual the editor’s comment highlights some papers. In the present issue we had 7 papers about prostate cancer (1–7) and we decided to comment the paper about a very interesting topic: The impact of Prostate Imaging-Reporting and Data System (PI-RADS) in Prostate Biopsy.

Doctor Rozas and collegues from Brazil performed on page 486 an interesting study about the impact of PI-RADS in prostate biopsy. The authors described the findings of multiparametric prostate resonance imaging (MRmp), parameterized with PI-RADS v2, using prostate biopsy as reference test and to assess the sensitivity and specificity of mpMR in identifying clinically significant prostate cancer using prostate biopsy as a reference test. They observed 342 patients with suspected prostate cancer that were evaluated with mpMR and prostate biopsy. The authors performed a total of 342 biopsies and concluded that mpMR is a useful tool to safely identify which patients at risk for prostate cancer need to undergo biopsy and has high sensitivity and specificity in identifying clinically significant prostate cancer.

Prostate cancer had important modifications in diagnosis, clinical management and surgical treatment in last years (8–11). Multiparametric magnetic resonance imaging (mpMRI) has become the standard of care and provides useful information for prostate cancer diagnosis (12). The Prostate Imaging-Reporting and Data System (PI-RADS) was created in 2012 to establish standardization in (mpMRI) acquisition, interpretation, and reporting of prostate cancer. In the presente paper Among the 83 patients with clinically significant tumor, 81 (97.5%) had positive MpMRp results and only 2 (2.5%) had a negative result. The great information of this paper is that all cancers (non clinically significant and clinically significant), the sensitivity of MpMRp was 85.5% and specificity 86.3%, with PPV of 80% and NPV of 90.5%.

We need more evidences, but we can conclude that the multiparametric prostate resonance imaging, parameterized with PI-RADS v2, using prostate biopsy will be the gold standard for the diagnosis of prostate cancer.
